# Supplementary material for: Modeling Coil–Globule–Helix Transition in Polymers by Self-Interacting Random Walks
Source: Polymers (Basel). 2023 Sep 7;15(18):3688. doi: 10.3390/polym15183688 (PMC10537616; doi:10.3390/polym15183688)
Supplement: Supplementary file 1 [file polymers-15-03688-s001.zip › polymers-2541174-supplementary.pdf]

Supplementary Materials

Modeling Coil-Globule-Helix Transition in Polymers by Self-Interacting Random Walks

Eddie Huang [1] and Zhi-Jie Tan[2,*]

[1] Wuhan Britain-China School, No.10 Gutian Ce Rd., Qiaokou District, Wuhan 430022, China
[2] School of Physics and Technology, Wuhan University, Wuhan 430074, China
* Correspondence: zjtan@whu.edu.cn

Key Words: Random walks, Statistical physics, Phase transition, Polymer, Helix

[*]Corresponding author: zjtan@whu.edu.cn

Supplementary Figure S1

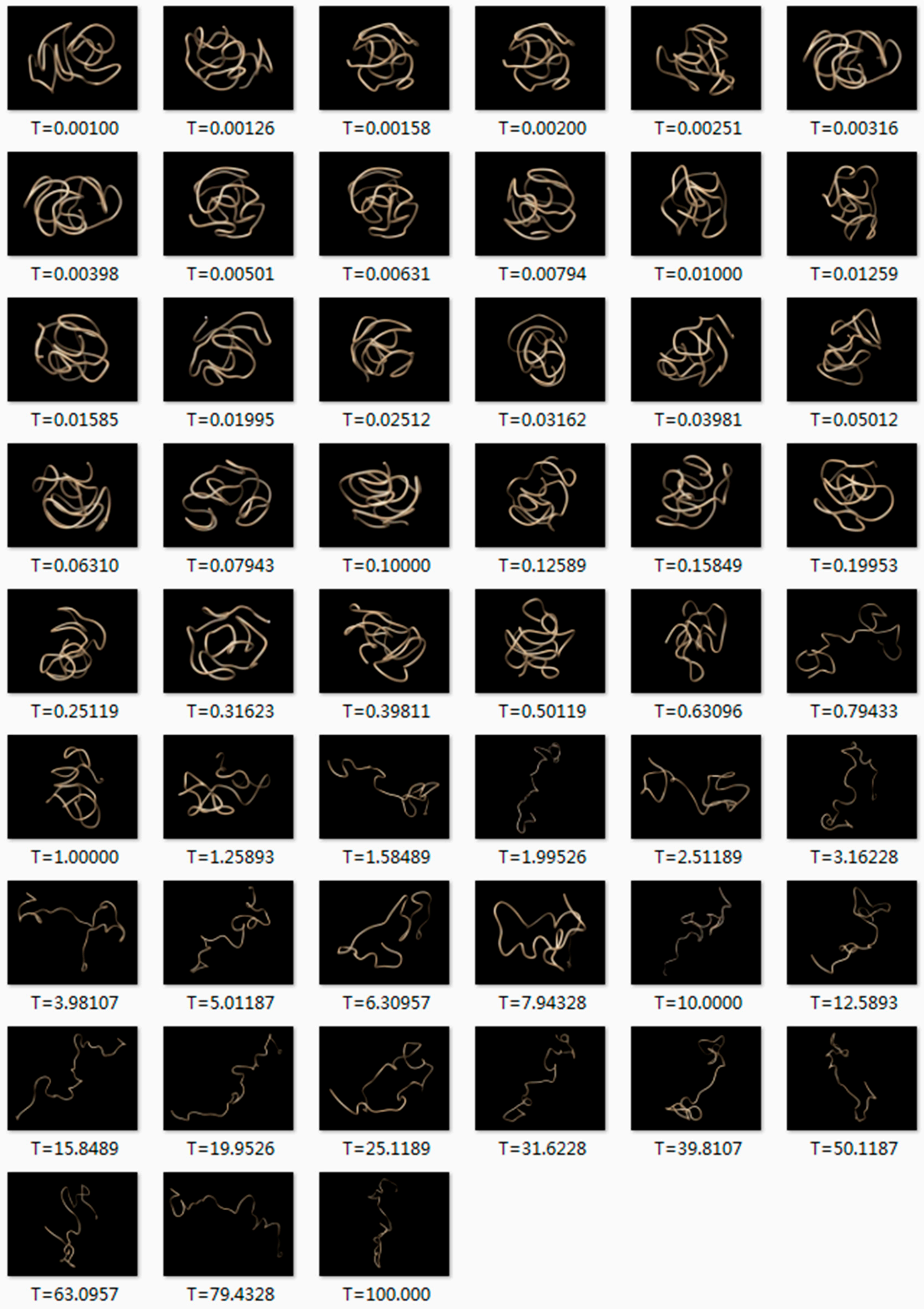

Supplementary Figure S1. Structures of the typical trajectories by self-interacting walks at different temperatures T when the equilibrium distance r_0 =1.00 and the well depth ε=1.0.

Supplementary Figure S2

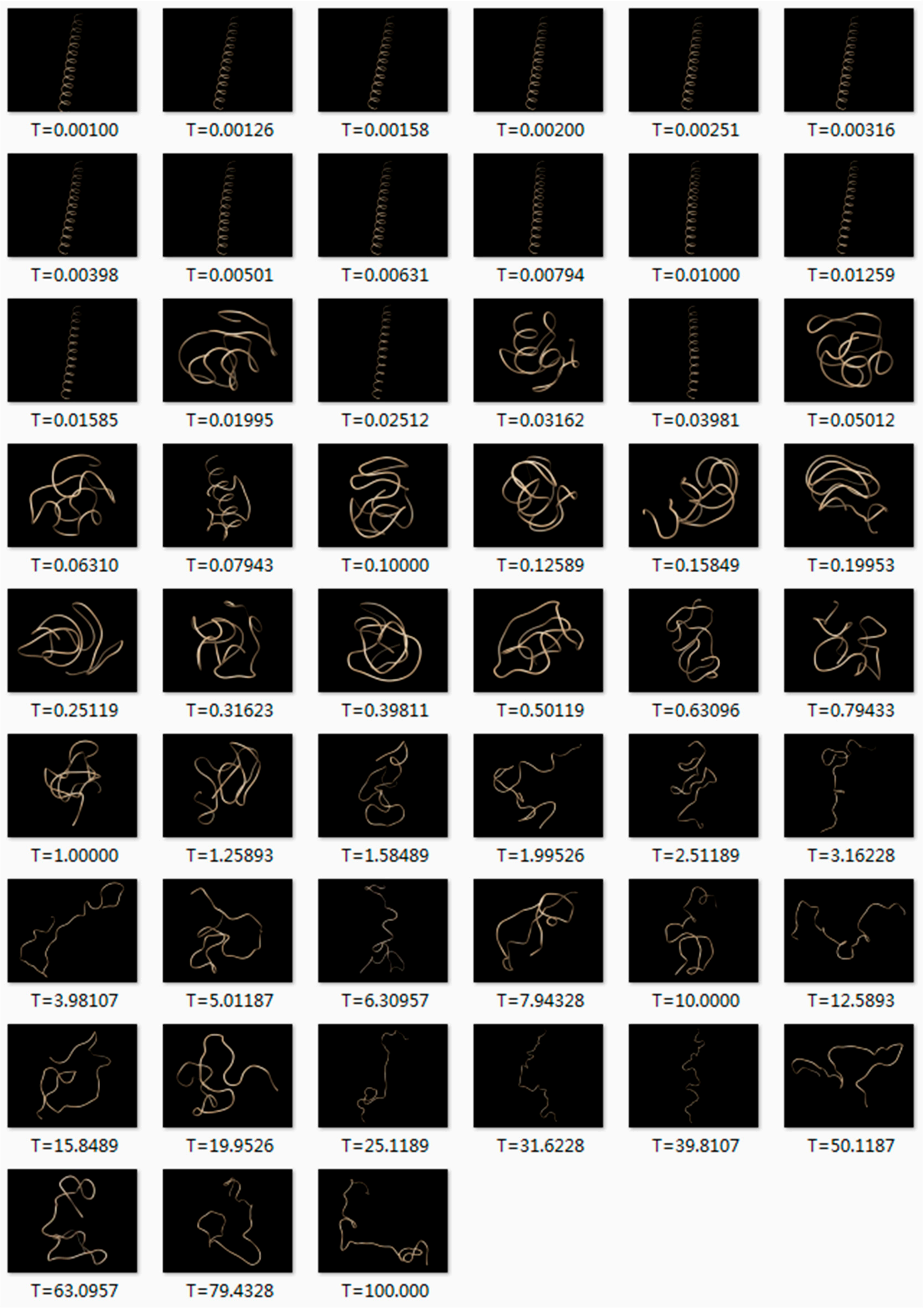

Supplementary Figure S2. Structures of the typical trajectories by self-interacting walks at different temperatures T when the equilibrium distance r_0 =1.52 and the well depth ε=1.0.

Supplementary Figure S3

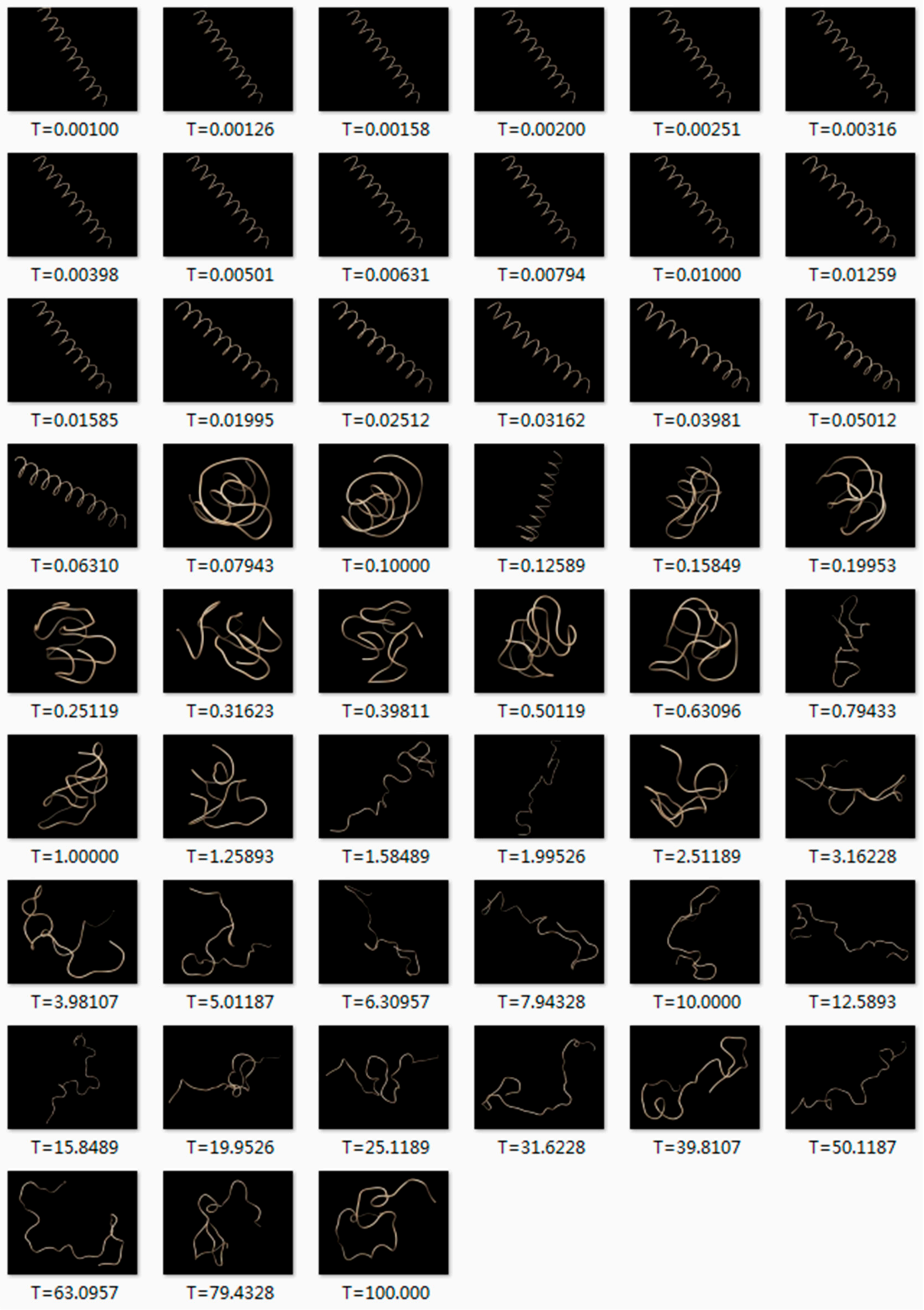

Supplementary Figure S3. Structures of the typical trajectories by self-interacting walks at different temperatures T when the equilibrium distance r_0 =1.69 and the well depth ε=1.0.

Supplementary Figure S4

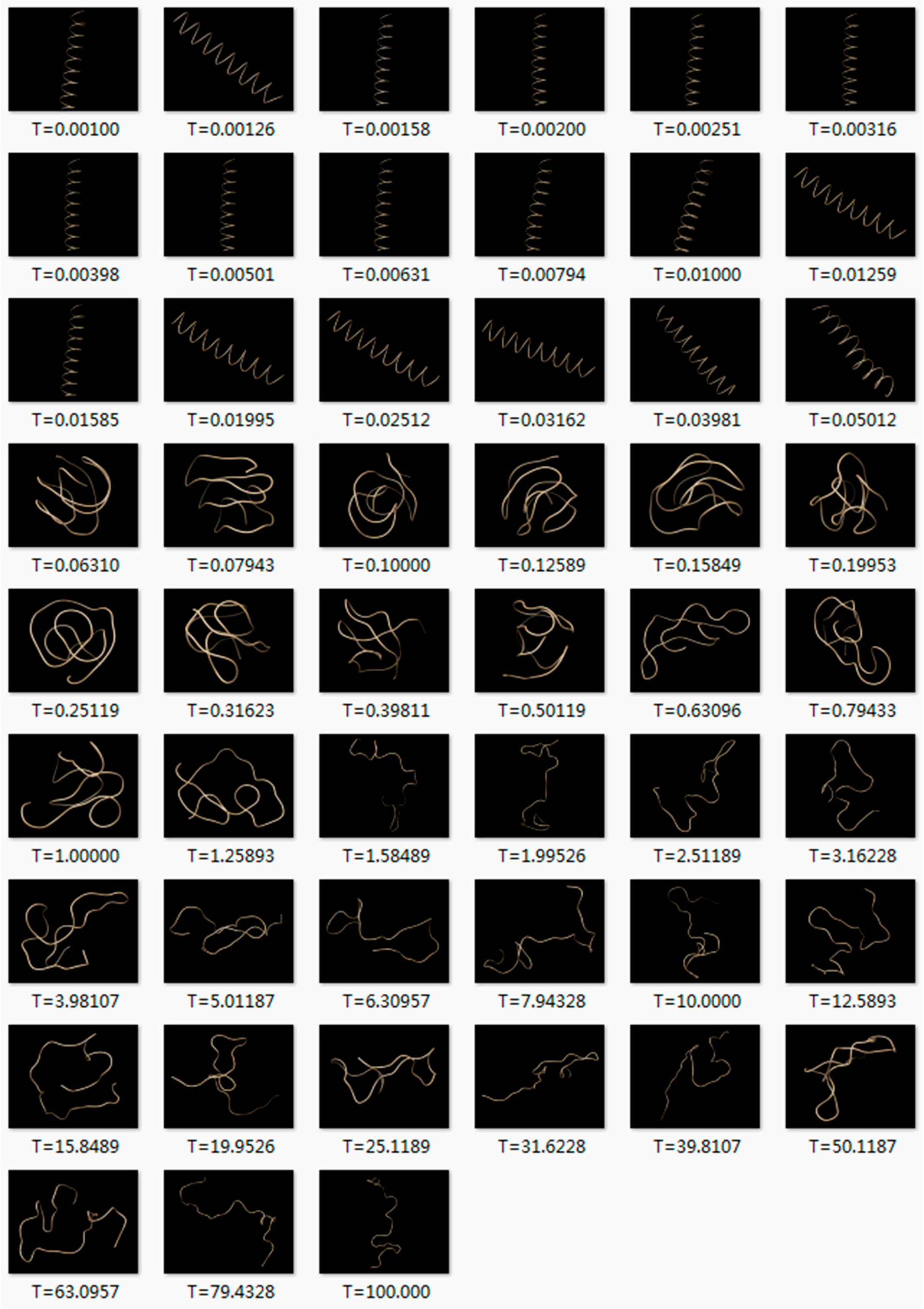

Supplementary Figure S4. Structures of the typical trajectories by self-interacting walks at different temperatures T when the equilibrium distance r_0 =1.82 and the well depth ε=1.0.

Supplementary Figure S5

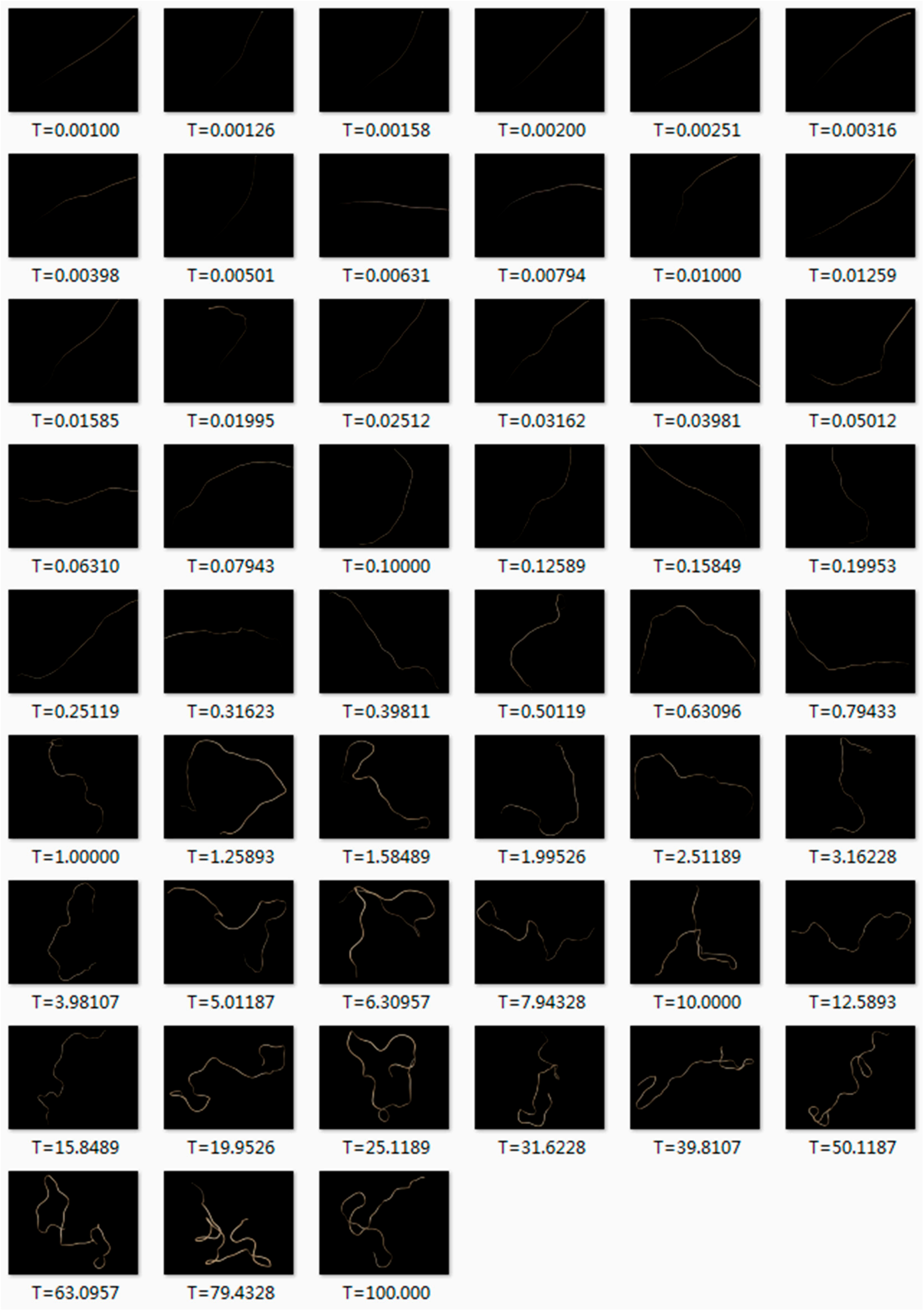

Supplementary Figure S5. Structures of the typical trajectories by self-interacting walks at different temperatures T when the equilibrium distance r_0 =2.25 and the well depth ε=1.0.
